# Cognitive Impairment in Chronic Obstructive Pulmonary Disease

**DOI:** 10.1371/journal.pone.0102468

**Published:** 2014-07-17

**Authors:** Alexandru F. Crişan, Cristian Oancea, Bogdan Timar, Ovidiu Fira-Mladinescu, Alexandru Crişan, Voicu Tudorache

**Affiliations:** 1 Department of Pulmonology, University of Medicine and Pharmacy “Victor Babes”, Timisoara, Romania; 2 Department of Biostatistics and Medical Informatics, University of Medicine and Pharmacy “Victor Babes”, Timisoara, Romania; 3 Department of Infectious Disease, University of Medicine and Pharmacy “Victor Babes”, Timisoara, Romania; Nathan Kline Institute and New York University School of Medicine, United States of America

## Abstract

**Background/Purpose:**

Chronic obstructive pulmonary disease (COPD), especially in severe forms, is commonly associated with multiple cognitive problems. Montreal Cognitive Assessment test (MoCA) is used to detect cognitive impairment evaluating several areas: visuospatial, memory, attention and fluency. Our study aim was to evaluate the impact of stable COPD and exacerbation (AECOPD) phases on cognitive status using MoCA questionnaire.

**Methods:**

We enrolled 39 patients (pts), smokers with COPD group D (30 stable and 9 in AECOPD) and 13 healthy subjects (control group), having similar level of education and no significant differences regarding the anthropometric measurements. We analyzed the differences in MoCA score between these three groups and also the correlation between this score and inflammatory markers.

**Results:**

Patients with AECOPD had a significant (p<0.001) decreased MoCA score (14.6±3.4) compared to stable COPD (20.2±2.4) and controls (24.2±5.8). The differences between groups were more accentuated for the language abstraction and attention (p<0.001) and delayed recall and orientation (p<0.001) sub-topics. No significant variance of score was observed between groups regarding visuospatial and naming score (p = 0.095). The MoCA score was significantly correlated with forced expiratory volume (r = 0.28) and reverse correlated with C-reactive protein (CRP) (r = −0.57), fibrinogen (r = −0.58), erythrocyte sedimentation rate (ESR) (r = −0.55) and with the partial pressure of CO_2_ (r = −0.47).

**Conclusions:**

According to this study, COPD significantly decreases the cognitive status in advanced and acute stages of the disease.

## Introduction

Chronic obstructive pulmonary disease (COPD) is a multi-component disorder that results in airflow limitation and respiratory distress and is frequently associated with a wide range of comorbidities and psychological problems. It is now well recognized that COPD patients suffer from many non-respiratory manifestations such as skeletal muscle dysfunction, systemic inflammation, nutritional depletion, malnutrition and neurological impairments. [1].

The occurrences of exacerbations adversely affect the natural course of the disease. An exacerbation of COPD is defined as an acute event characterized by worsening of the patient’s respiratory symptoms that is beyond normal day-to-day variations and leads to a change in medication, dyspnea and/or volume/color of sputum, need of antibiotic treatment, or need of hospitalization [2] One of the negative effects of exacerbation is the cognitive alteration.

A mechanism proposed for the cognitive impairment in COPD patients is the neuronal damage mediated by hypoxia as a result of the pulmonary disease or the comorbidities that adversely affect the brain, such as vascular disease and smoking [3].

Furthermore, it has been suggested that the low performance in neuropsychological test can be a predictor of mortality and disability in certain COPD populations [4].

Mild cognitive impairment, also known as incipient dementia, is defined as a clinical condition characterized by the decline of cognitive function greater than expected for a certain age and educational level of the individual but not severe enough to interfere with their daily activities [5].

Poor relevant markers of patient outcomes, such as airflow limitation, do not reflect the multi-system nature of the disease. Therefore, it is important to identify, assess and understand the relevant comorbidities in COPD to better characterize the full clinical spectrum of the disease and cognitive impairment is one of the proposed comorbidities [6].

The Montreal Cognitive assessment test is an efficient instrument to use for screening, diagnosis and tracking of mild cognitive impairment (MCI). It assesses different cognitive domains, has good psychometric properties and has become a widely used screening instrument for MCI. [7] It was created in response to the poor sensitivity of the Mini-Mental State Examination (MMSE) for distinguishing patients with mild cognitive impairment from normal elderly patients [8].

## Purpose of the Study

The aim of our study is to evaluate the cognitive impact of COPD using MoCA test and to observe how the inflammation is influencing cognition.

## Materials and Methods

The ethical committee of the University Hospital of Pulmonology and Infectious Disease “Dr. Victor Babes”, Timisoara approved this study. Prior participation all patients signed an inform consent form.

Montreal Cognitive Assessment is a 30 point test that takes between 10 to 15 minutes to complete and assesses several cognitive domains. A score below 26 points shows a mild cognitive impairment. It assesses visuospatial abilities using a clock drawing task (3 points), and a three-dimensional cube copy (1 point). Multiple aspects of executive functions are assessed using a trail-making task (1 point), a phonemic fluency task (1 point), and a two-item verbal abstraction task (2 points). Language is assessed using a three item confrontation naming task with familiar animals (3 points) and repetition of two syntactically complex sentences (2 points). Short term memory is evaluated with a task that involves two learning trials of five nouns and delayed recall after approximately 5 minutes (5 points). Attention, concentration and working memory are evaluated using a sustained attentions task (target detection using tapping, 1 point), digits forward and backward (1 point each) and a serial subtraction task (3 points). At the end of the test, orientation and place is assessed (6 points).

### Subjects

We performed a cross-sectional, observational study. The individuals from the stable and AECOPD groups were selected from the patients admitted in the Department of Pulmonology, University Hospital “Dr. Victor Babes” in a population-base consecutive enrollment process, while the control group was composed by healthy volunteers.

At the beginning of the study, 17 healthy subjects and 72 COPD patients were enrolled.

The inclusion criteria for healthy subjects was age between 40 and 80 years. For stable COPD patients: GOLD class D without exacerbation of respiratory system in the past two months.

Exclusion criteria for stable COPD and control subjects: refusal to participate, age <40 and >80 years, illiteracy, alcoholism, obesity, long term oxygen therapy, severe cardiovascular comorbidities (refractory hypertension to medical treatment, heart failure New York Heart Association class III/IV, myocardial infarction, congestive heart failure), cancer, uncontrolled diabetes, major cognitive impairment, history of head injury or brain tumor, dementia, epilepsy.

37 pts. drop-outs were registred, being excluded from the statistical analysis: 4 healthy subjects from the control group due to alcoholism (1 pts.) and illiteracy (3 pts.), 20 pts. from stable COPD group (4 pts. long term oxygen therapy, 3 pts. alcoholism, 2 pts. illiteracy, 4 pts. diabetes, 2 pts. refusal to participate, 2 pts. refractory hypertension to medical treatment, 1 pts. with lung cancer, 2 pts. therapy with betahistine dihydrochloride) and 13 pts. from the AECOPD group (1 pts. illiteracy, 2 pts. obesity and obstructive sleep apnea syndrome, 2 pts. myocardial infarction, 4 pts. non-invasive ventilation, 1 pts. dementia, 2 pts. history of head injury, 1 pts. therapy with alprazolam). Fifty-two subjects remained to be investigated (control group 13 subjects, stable COPD group 30 pts. and 9 pts. in the AECOPD group). The COPD patients met the ATS/ERS criteria for COPD risk class D. All patients presented similar anthropometric parameters and had similar education level. On admission day all the patients performed spirometry using the Medgraphics device as well as blood samples and gases were collected. All patients completed the MoCA questionnaire in the first day of hospitalization. The completion of the questionnaire was conducted and supervised by the same physiotherapist.

### Statistical analysis

Data was collected and analyzed using SPSS v.17.0 (SPSS Inc, Chicago, IL, USA) and is presented as mean ± standard deviations in case of continuous variables with Gaussian distribution, median and interquartile range for continuous variables without Gaussian distribution or percentages for categorical variables.

To assess the significance of the differences between groups, the ANOVA along with Bonferroni post-hoc (means, Gaussian populations), Kruskal-Wallis and Mann-Whitney-U (medians, non-Gaussian populations) tests were used. Continuous variables distributions were tested for normality using Shapiro-Wilk test (if the p value was higher than 0.05, Gaussian distribution was assumed) and for homoscedasticity using Levene’s test. The strength of correlations between variables was assessed using Spearman’s correlation coefficient and its statistical significance with t-distribution test. In order to evaluate the involvement of multiple confounding variables on the MOCA score, a multivariate regression model was built, having as outcome the MOCA score. The predictors were added in the model using a forward-stepwise method, the principle of adding or removing one term from the regression equation being based on the information entropy principle; it was chosen the model having the lowest corrected Akaike’s Information Criterion score.

A p value of <0.05 was considered the threshold for statistical significance.

## Results

**Figure 1:**
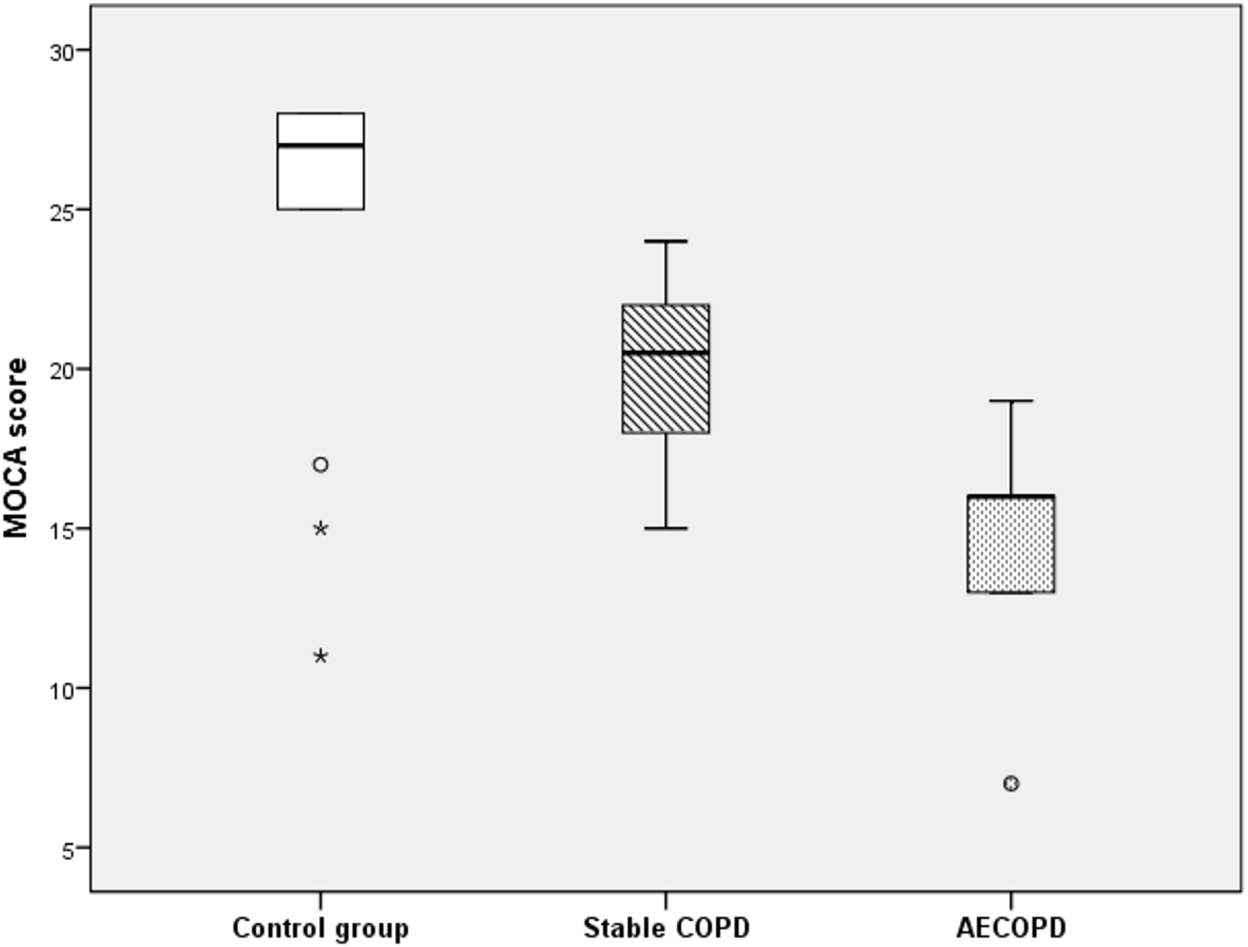
BoxPlot for MoCA score in the three study subgroups. The band inside the box represents the median score; the top and bottom of the box the third respectively the first quartile; the whiskers - data within 1.5 interquartile range and stars and circles the outliers.

The MoCA scores were Gaussian distributed in the entire group (Shpairo-Wilk’s p = 0.21) and also for all the three subgroups. We observed that in the group of patients with COPD exacerbations the MoCA score was significantly lower compared to the one recorded in patients with stable COPD and controls, despite having no significant differences in age (Figure 1).

The decrease trend in MoCA score was significant for the global score (p<0.001) and also for two sub-topics of the questionnaire: language, abstraction and attention (p<0.001) and delayed recall and orientation (p<0.001), while the differences regarding visual-spatial and naming score had no statistical significance (p = 0.095). The presence of COPD exacerbations were associated with increased fibrinogen (4.5 vs. 2.9, p<0.001) and CRP (33.5 vs. 5.9, p = 0.017), when compared to patients with stable COPD. No significant differences were observed between these two groups regarding the average number of red blood cells (RBC) and white blood cells (WBC), PaO_2_, PaCO_2_, pH, FEV_1_ and FVC (Table 1).

**Table 1:** Comparison of patients parameters between studied groups.

Parameter	Controls (A)	Stable COPD (B)	AECOPD (C)	p-value	
				A vs. B vs. C	B vs. C
MOCA score^a^	24.2±5.8	20.2±2.4	14.6±3.4	<0.001	0.001
Visual-spatial and naming (max. 8 pts.)^a^	6.3±0.7	6.1±1.0	5.0±1.3	0.095	0.156
Language, abstraction and attention (max. 11 pts.)^a^	10.5±1.1	6.8±1.6	4.4±1.3	<0.001	0.006
Delayed recall and orientation (max. 11 pts.)^a^	9.5±0.5	7.1±1.4	5.8±1.6	<0.001	0.023
Age (years)^b^	58 [15]	60 [12]	67 [12]	0.052	0.123
Fibrinogen^a^	n/a	2.9±0.7	4.5±1.4	n/a	<0.001
CRP^a^	n/a	5.9±7.5	33.5±27.4	n/a	0.017
RBC^a^	n/a	4.9±0.5	5.2±1.0	n/a	0.370
WBC^a^	n/a	7.8±1.9	8.3±3.1	n/a	0.346
FVC (%)^a^	98.4±14.1	48.8±15.1	46.4±16.5	<0.001	0.998
FVC (L)^a^	4.2±1.2	2.3±0.7	1.7±0.8	<0.001	0.328
FEV_1_ (%)^a^	92.9±8.9	23.7±5.1	25.9±6.6	<0.001	0.988
FEV_1_ (L)^a^	3.2±0.9	0.8±0.2	0.7±0.3	<0.001	0.964
PaO_2_ a	n/a	70.3±8.4	71.8±11.7	n/a	0.705
PaCO_2_ a	n/a	42.1±3.7	105.4±73.7	n/a	0.306
pH^a^	n/a	7.40±0.02	7.41±0.03	n/a	0.520

a Variables are Gaussian distributed. Results are presented as mean ± standard deviation. p values were calculated with unpaired t-student and ANOVA tests.

b Variables are not-Gaussian distributed. Results are presented as median and interquartile range. p values were calculated using Kruskal-Wallis and Mann-Whitney U tests.

CRP – C-reactive protein, RBC – Red blood cell, WBC – White blood cell, FVC – Force vital capacity, FEV_1_ – Forced expiratory volume.

At the univariate regression analysis, in our studied group the results of the MoCA score were reverse and significantly correlated with CRP, fibrinogen, ESR, pCO_2_ and it was positively correlated with the FEV_1_ (%) value. The direct correlation between pO_2_ and MoCA score had no statistical significance (Table 2).

**Table 2:** Correlation between MOCA score and clinical parameters.

Correlations		FEV (%)	CRP	Fibrinogen	ESR	PaO_2_	PaCO_2_
MOCA	Spearman r – entire group	0.28	−0.57	−0.58	−0.55	0.25	−0.47
	P	0.044	0.001	0.001	0.001	0.178	0.007

The resulted multivariate regression model (r = 0.60; p = 0.016), having as outcome the MoCA score revealed significant influence for: FEV_1_ (positive correlated), fibrinogen (inverse correlated) and ESR (reverse correlated) (Table 3). In the model was also included CRP, but it was not significant correlated, other possible predictors being excluded by the increase in Akaike’s score (age, BMI, PaO_2_, PaCO_2_, pH and FVC).

**Table 3:** Multivariate regression analysis between studied parameters and MoCA score.

Predictor	Exp(β)	P
FEV_1_	0.374	0.017
Fibrinogen	−0.350	0.043
CRP	−0.143	0.326
ESR	0.262	0.290

## Discussion

Most people, over their life span, suffer of a gradual cognitive impairment, usually regarding memory. Typically the decline is minor, and despite being unpleasant for the patients, it does not compromise the ability to function normally.

Mild cognitive impairment is classified into two subtypes: amnestic and non-amnestic. The amnestic type comes with significant memory impairment but does not meet the criteria for dementia. Other cognitive functions such as use of language, visuospatial skills and executive function are relatively preserved. Non-amnestic mild cognitive impairment is characterized by a subtle decline in functions not related to memory, affecting attention, use of language, or visuospatial skills [9].

The cognitive impairment is a significant concern for elderly because it can decrease quality of life and, in advance stages, it might cause functional disabilities.

To our knowledge this is the first study which uses MoCA questionnaire to assess the cognition in patients with AECOPD. Searching the literature for the best questionnaire we had chosen MoCA instead of the MMSE because of the lower sensitivity of the later one. MoCA can be more helpful in classifying patients in the borderline area between MCI and dementia than MMSE. We think that MOCA may be better suited than MMSE in detecting the earliest stages of impairment. Dong Y, and Sylvia Villeneuve compared the questionnaires and concluded that MoCA is superior to MMSE in detection of patients with cognitive impairment [10], [11].

Studies mentioned that adults with COPD are having an increased rate of cognitive impairment, especially those who require oxygen therapy [12].

Literature regarding hypoxia influences on the cognitive function in patients with COPD are pointing to minor cognitive impairment MCI with decline in attention, slower mental speed and compromised executive functions [13].

Compared to a study that has observed cognitive impairment in 77% of patients with both COPD and hypoxemia, we found that all our COPD patients have a minor cognitive impairment although they had a borderline hypoxemia [14].

Specific patterns of cognitive deterioration, characterized by a dramatic impairment in verbal memory tasks, diffuse worsening of other functions and well preserved visual attention where shown by Incalzi el al. in 48.5% of patients with COPD [15].

Compared to Incalzi’s study, we observed a significant decrease in abstraction, attention and delayed recall in our COPD patients especially in the exacerbation group. CO_2_ retention is a problem in respiratory diseases, especially in COPD so we may assume that because of this retention and systemic inflammation, our COPD patients have a decreased cognitive function. However, further research is needed to sustain this theory.

Aging is accompanied by increased immune dysregulation. IL-6 and other cytokines may play an important role in the chronic inflammation and neurodegeneration characteristic of the cognitive decline. [16] Circulating markers of inflammation, including fibrinogen, were associated with a significant faster 4 year decline in general cognition [17].

Similar to the study conducted by Snorri, we observed that fibrinogen was higher in those patients who had a lower MoCA score thus inferring indirectly that high levels of fibrinogen could lead to a decreased cognitive function. Since there are some other factors that can influence cognition, we cannot point only at this inflammatory marker.

In a study which evaluated over 4000 subjects it was observed that a higher level of CRP and IL-6 was associated with decreased cognition and executive function [18].

In our study we observed that patients who had a high level of CRP had a lower MoCA score. The authors of another study concluded that relatively high concentrations of CRP can be an indicative for impaired cognitive performance [19].

It is well known that the most important risk factor that leads to COPD is smoking. All our COPD patients in this study were smokers, so we assume that the inflammatory cytokines released over the years because of the smoke may lead to synaptic alteration and so, the destruction on existing neurons, led to a decrease of cognitive function.

Chronic systemic inflammation induced by stimuli such as cigarette smoking, obesity, disrupted sleep patterns compromises the integrity of the brain-blood barrier allowing irritants to enter the brain and stimulate the production of inflammatory cytokines. The cytokines impair neurogenesis and synaptic transmission and aside that, some inflammatory cytokines damage and destroy existing neurons by stimulating apoptosis [20].

Observational data have shown associations between the presence of cardiovascular risk factors in patients with MCI and an increased risk of progression to dementia [21].

Lung function is another mechanism that could underlie the association between smoking and cognitive decline. Smoking is a risk factor for lung injuries that can increase the risk of cognitive impairment and dementia. [22] We observed that low FEV_1_ was associated with decreases in MoCA score.

Compared to never smokers, middle-age male smokers are likely to experience faster 10-year cognitive decline in executive function and global cognition [23].

## Conclusions

From our results we can conclude that patients with COPD are having significant impairment of the cognitive status, especially in advanced stages of the disease and after exacerbation episodes are occurring.

Increased inflammatory markers in patients with COPD are associated with the decrease of the cognitive functions, this relationship being caused by a series of mechanisms that need further research to be completely understood.
